# Candidate loci shared among periodontal disease, diabetes and bone density

**DOI:** 10.3389/fendo.2022.1016373

**Published:** 2023-01-27

**Authors:** Yau-Hua Yu, Bjorn Steffensen, Paul M. Ridker, Julie E. Buring, Daniel I. Chasman

**Affiliations:** ^1^ Department of Periodontology, Tufts University School of Dental Medicine, Boston, MA, United States; ^2^ Division of Preventive Medicine, Brigham and Women’s Hospital, Boston, MA, United States; ^3^ Harvard Medical School, Boston, MA, United States

**Keywords:** genetics, oral health, diabet mellitus, periodontal disease, bone densities, GWAS, survey and questionnaire

## Abstract

**Introduction:**

While periodontal disease (PD) has been associated with type 2 diabetes (T2D) and osteoporosis, the underlying genetic mechanisms for these associations remain largely unknown. The aim of this study is to apply cross-trait genetic analyses to investigate the potentially shared biology among PD, T2D, and bone mineral density (BMD) by assessing pairwise genetic correlations and searching for shared polymorphisms.

**Methods:**

We applied cross-trait genetic analyses leveraging genome-wide association study (GWAS) summary statistics for: Periodontitis/loose teeth from the UKBB/GLIDE consortium (PerioLT, N=506594), T2D from the DIAGRAM consortium (N_eff_=228825), and BMD from the GEFOS consortium (N=426824). Among all three, pair-wise genetic correlations were estimated with linkage disequilibrium (LD) score regression. Multi-trait meta-analysis of GWAS (MTAG) and colocalization analyses were performed to discover shared genome-wide significant variants (p_MTAG_ <5x10^-8^). For replication, we conducted independent genetic analyses in the Women’s Genome Health Study (WGHS), a prospective cohort study of middle-aged women of whom 14711 provided self-reported periodontal disease diagnosis, oral health measures, and periodontal risk factor data including incident T2D.

**Results:**

Significant genetic correlations were identified between PerioLT/T2D (Rg=0.23; SE=0.04; p=7.4e^-09^) and T2D/BMD (Rg=0.09; SE=0.02; p=9.8e^-06^). Twenty-one independent pleiotropic variants were identified *via* MTAG (p_MTAG_<5x10^-8^ across all traits). Of these variants, genetic signals for PerioLT and T2D colocalized at one candidate variant (rs17522122; Prob_H4 =_ 0.58), a 3’UTR variant of *AKAP6*. Colocalization between T2D/BMD and the original PerioLT GWAS p-values suggested 14 additional loci. In the independent WGHS sample, which includes responses to a validated oral health questionnaire for PD surveillance, the primary shared candidate (rs17522122) was associated with less frequent dental flossing [OR(95%CI)= 0.92 (0.87-0.98), p=0.007], a response that is correlated with worse PD status. Moreover, 4 additional candidate variants were indirectly supported by associations with less frequent dental flossing [rs75933965, 1.17(1.04-1.31), p=0.008], less frequent dental visits [rs77464186, 0.82(0.75-0.91), p=0.0002], less frequent dental prophylaxis [rs67111375, 0.91(0.83-0.99), p=0.03; rs77464186, 0.80(0.72-0.89), p=3.8e^-05^], or having bone loss around teeth [rs8047395, 1.09(1.03-1.15), p=0.005].

**Discussion:**

This integrative approach identified one colocalized locus and 14 additional candidate loci that are shared between T2D and PD/oral health by comparing effects across PD, T2D and BMD. Future research is needed to independently validate our findings.

## Introduction

Periodontal disease (PD) is a highly prevalent microbial induced chronic inflammatory disease with variable clinical expression. It has been estimated that 46% of US adults had periodontitis with 8.9% having severe periodontitis (1). PD is known to be associated with innate and adaptive immune responses (2), genetic susceptibility factors (3, 4), type 2 diabetes (T2D) (5), and osteoporosis (6). It has been shown that advanced PD progression correlated with poor glycemic control (7). Conversely, improvement in glycemic control among diabetic patients (8, 9) and reduction of healthcare expenditures (10, 11) were reported after periodontal treatment. Recent reviews of bone diseases among diabetic patients further shed light on the complex biology between diabetes, bone pathophysiology and microvasculature (12).

Associations between PD and T2D suggest overlapping etiology, but specific shared genetic mechanisms remain largely unknown, despite significant genetic correlation (13). Such mechanisms may be intrinsically difficult to discover due to complexity in the diversity of additional highly correlated comorbidities such as osteoporosis, hypercholesterolemia, and metabolic traits (14). Specifically, progress has been hampered in part by the lack of significant genetic risk variants and therefore implicated biology from the GWAS of PD, a situation that is in sharp contrast with the GWAS of T2D, which has abundant significant signals. Numerous candidate PD variants have been reported over the past decade, albeit with most studies reporting a sub-genome-wide level of statistical significance when using various PD definitions (15). Indeed, performing clinical periodontal examination in a large epidemiological study has been challenging and resource demanding (16). Thus, in the setting of GWAS that require large samples, PD has been defined with various alternative and possibly incommensurate approaches (e.g., various case definitions with different clinical measures or treatment histories) and/or self-reported responses (13). Even with sample sizes as large as 506594 (*n*_cases_=36150), identification of genetic factors for PD has not been successful, likely in part due to heterogenous disease definitions (13). To standardize PD measures in epidemiological surveillance and research, the Centers for Disease Control and Prevention (CDC) and the American Academy of Periodontology (AAP) together created and validated a list of oral health questions (OHQs) for assessing periodontal status (17, 18). Participants who responded with OHQs indicating less-than-ideal oral health behaviors such as less frequent interdental cleaning were more likely to have severe PD (19). Lack of routine dental care was also shown to predict tooth loss (20). Selected CDC-AAP OHQs have recently been adopted in large genomic databases such as the UK Biobank (13), the Million Veterans Program (21) and the Women’s Genome Health Study (WGHS) (22) whose oral health status in 2018 was obtained using established CDC-AAP OHQs.

In this study, to overcome difficulties in identifying significant genomic loci for PD, we explored potential shared genetics of the large scale GWAS summary statistics for periodontitis/loose teeth (PerioLT) from the UK Biobank/GLIDE (13), for T2D from the DIAGRAM consortium (23), and for bone mineral density (BMD) from the GEFOS consortium (24) using genetic correlation analysis (25) and multi-trait analysis of GWAS (MTAG) meta-analysis (26) followed by genetic colocalization analysis of candidate loci (27). The approach used here has potential to overcome challenges from the intrinsic heterogeneity in PD definitions by focusing statistical power on loci by PerioLT and either T2D or BMD, or both. If there are weak, sub-genome-wide significant (p > 5x10^-8^) genetic signals in the published PerioLT GWAS that are nevertheless shared by other conditions, as had been suggested for PerioLT and T2D in the prior analysis (13), then MTAG provides a way to enhance these genetic signals by leveraging genome-wide genetic correlation with the other traits. In addition, to further characterize the candidate loci, we evaluated their associations with specific measures of periodontal status in the WGHS collected through OHQs.

## Material and methods

### GWAS summary statistics for periodontitis/loose teeth, type 2 diabetes, and bone mineral density

Summary statistics were publicly available from the GWAS consortia for the meta-analyses of case-control GWASs of periodontitis/loose teeth (abbreviated as PerioLT, downloaded from the UKBB/GLIDE, https://data.bris.ac.uk/data/dataset/7276c102292c49d4098a8c4396849218) (13), for the meta-analyses of case-control GWASs of type 2 diabetes (abbreviated as T2D, downloaded from the DIAGRAM consortium, https://diagram-consortium.org/downloads.html) (23), and for the meta-analyses of continuous heel bone mineral density (abbreviated as BMD, downloaded from the GEFOS consortium, http://www.gefos.org/?q=content/data-release-2018) (24).

### Study population of the Women's Genome Health Study

Participants in the WGHS were initially healthy, female healthcare professionals at least 45 years of age at baseline and represented participants in the Women’s Health Study (WHS) who provided a blood sample at baseline. The WHS was conducted as a two-by-two randomized clinical trial in 1992-1994 investigating the effects of vitamin E and low dose aspirin in prevention of cancer and cardiovascular diseases with 10 years of follow-up (22, 28). Since the end of the trial, follow-up has continued in an observational mode. Additional information related to health and lifestyle were collected by questionnaires throughout the WHS trial and continuing observational follow-up. The WHS/WGHS was approved by the Institutional Review Board of Brigham and Women’s Hospital, Boston Massachusetts and this report conforms to the STROBE guidelines (29).

### Self-reported oral health questions in the WHS/WGHS

Information on the CDC-AAP validated OHQs (18) were collected in 2018 when WHS/WGHS participants were asked the questions that are provided in **Box 1**. These questions were administered to assess periodontal health. By December 2018, a total of 17955 questionnaires with information about updated oral/periodontal health were obtained. Among these, 14663 WGHS women of verified European ancestry had complete information for genotype, OHQs, and T2D diagnosis ascertained in December 2017.

Box 1 **Self-reported Oral Health Questions (OHQs)**

**Table:** 

No.	Question
1	Do you think you might have gum disease?
	O Yes O No O Don’t know
2	Overall, how would you rate the health of your teeth and gums?
	O Excellent O Very good O Good O Fair O Poor O Don’t know
3	Have you EVER had treatment for gum disease such as scaling and root planing, sometimes called “deep cleaning?”
	O Yes O No O Don’t know
4	Have you EVER been told by a dental professional that you lost bone around your teeth?
	O Yes O No O Don’t know
5	Aside from brushing your teeth with a toothbrush, in the LAST 7 DAYS, on how many DAYS did you use dental floss or any other device to clean between your teeth?
	O 0 O 1 O 2 O 3 O 4 O 5 O 6 O 7
6	In the PAST 12 MONTHS, have you visited a dentist or dental hygienist?
	O Yes O No O Don’t know
7	How often do you usually visit the dental office for routine check-ups or cleanings?
	O More than once per year O Once per year O Less than once per year O Don’t know

### WGHS/WHS covariates and the ascertainment of type 2 diabetes or osteoporosis

In the WGHS, covariates such as age, race, education, income, body-mass-index, histories of hypercholesterolemia or hypertension, and smoking behaviors were summarized from the baseline study entry questionnaire. Observational health outcomes such as cardiovascular disease, diabetes, and cancers were followed up yearly by questionnaires and validated by medical records as previously reported (28, 30, 31). In this analysis, we used the 2009 self-reported diagnosis of osteoporosis, which was also confirmed based on participants’ report of having a bone density scan.

### Genetic data in the WGHS

Genotyping and imputation in the WGHS have been detailed in previous reports (22). Genotyping was performed on the HumanHap300 Duo array or the combination of the HumanHap300 Duo and iSelect arrays (Illumina, San Diego, CA) with the Infinium II protocol. Imputation of genotypes for SNPs that were not directly measured by the arrays was performed using genotyped SNPs that passed a test of HWE (p-value > 10^-6^) but were unrestricted by MAF, using the 1000G (phase 3, version 5) ALL panel (32) with MaCH (v.1.0.16) and Minimac. The majority of the WGHS participants have European ancestry verified with multidimensional scaling analysis using informative markers in PLINK. For this report, we used data from 14663 individuals of verified European ancestry who also had available information for type 2 diabetes and responses to the OHQs.

### Genetic methods and statistical analyses

Genome-wide genetic correlation was estimated using LD score regression (LDSC, v.1.0.1, https://github.com/bulik/ldsc) (25) using the reference panel provided with the software. MTAG (v1.0.8, https://github.com/JonJala/mtag) was performed using default settings (26). Analysis was restricted to variants with MAF >0.01. Colocalization analysis was performed with the R function coloc (v.5.1.0) using default settings (27). Coloc evaluates the posterior probability of 4 alternative hypotheses within the local interval around an index variant: H1= causal variant for trait 1 only; H2= causal variant for trait 2 only; H3= two distinct causal variants; H4= one common causal variant; with the null hypothesis of H0 = no causal variant. Multi-clumping was done using PLINK (v1.9, www.cog-genomics.org/plink/1.9/) with the 1000 genomes European LD reference panel (v.3). In summarizing demographic characteristics, group means and proportions were compared by t-tests for continuous variables and by chi-square tests for categorical variables, respectively, using R statistical software. Statistical significance was judged by p<5x10^-8^ for genome-wide analyses such as MTAG, by unadjusted two-sided p<0.05 for association analyses with clinical, i.e. non-genetic variables, or by p<3x10^-3^ for genetic associations of the 15 candidate MTAG SNPs with OHQ responses in the WGHS to account multiple testing.

As described above, the CDC-AAP OHQs were designed for PD surveillance in settings where direct clinical periodontal evaluation is not feasible, such as the WGHS. In this report, we note that we are therefore testing genetic associations of candidate loci shared between T2D and PerioLT where the responses to the OHQs in the WGHS serve as a proxy for the latter.

### Study flow diagram

**Figure 1:**
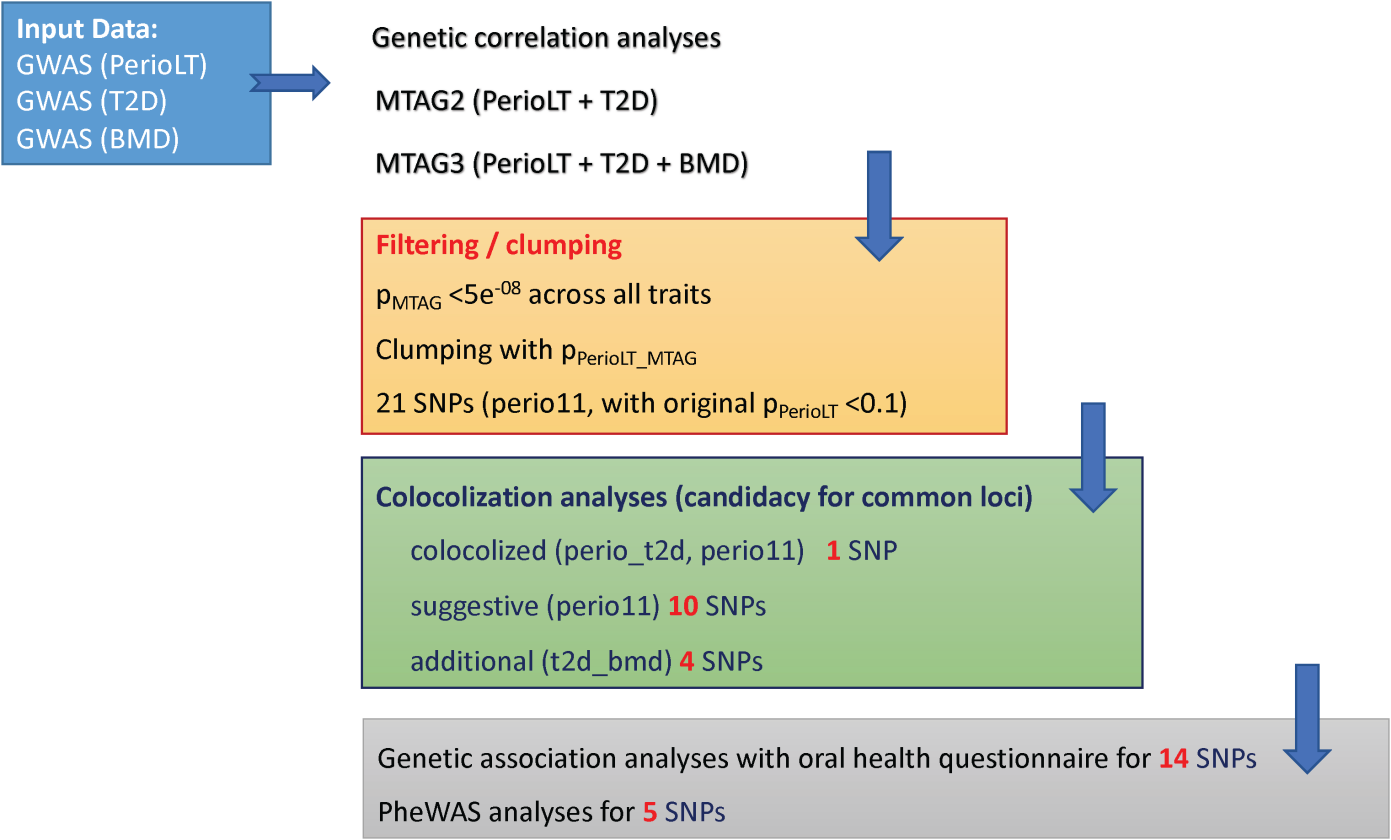
Study flow chart describing the steps in the analyses (1). Multi-trait meta-analysis of GWAS (MTAG) was conducted between PerioLT vs. T2D as well as among PerioLT/T2D/BMD (2). Filtering by the MTAG p-values <5x10^-8^ for all three traits and subsequently clumping by the PerioLT MTAG p-values, we identified 21 candidate variants (3). Among them, colocalization analyses suggested 15 shared loci (4). Genetic association analyses for 14 candidate variants were done in the independent WGHS sample *via* a validated oral health questionnaire for periodontal disease surveillance. Phenome-wide association study (PheWAS) was conducted for 5 loci demonstrating independent significant associations with additional, i.e. non-periodontal, phenotypes.

A flow diagram is provided in **Figure 1** to summarize the steps of analyses in this report.

## Results

### Genome-wide genetic correlations between periodontal disease, type 2 diabetes and bone mineral density

We estimated the genome-wide genetic correlations among GWASs of PerioLT (13), T2D (23), and BMD (24) using LDSC (**Table 1**) (25). PerioLT GWAS was significantly correlated with T2D [r_g_(SE)= 0.23 (0.04); p=7.4e^-09^], but there was no significant genetic correlation between PerioLT and BMD [r_g_(SE)= -0.02 (0.04); p=0.57]. There was a significant genetic correlation between GWAS of T2D and BMD [r_g_ (SE)= 0.09 (0.02); p=9.8e^-06^]. Such a finding has been suggested in an earlier report (12), but not evaluated on a genome-wide basis until the present investigation. The direction of the genetic correlation indicates that increased risk of T2D is associated with higher BMD. Our results suggest that a genetic relationship between PerioLT and BMD was not supported, despite the fact that many epidemiological reports suggested strong associations between PD and osteoporosis (6, 33). Moreover, in light of the significant genetic correlations between T2D/BMD in an opposite direction with that of PerioLT/BMD, it may be that alveolar bone loss due to PD is not genetically the same as those measured in heel BMD under the osteoporotic condition.

**Table 1:** Genome-wide genetic correlations of GWASs of periodontitis/loose teeth (PerioLT), type 2 diabetes (T2D), and bone mineral density (BMD).

	PerioLT		T2D	
GWAS summary stats	r_g_ (SE)	Pv	r_g_ (SE)	Pv
Periodontitis/loose teeth	1	**-**	**-**	**-**
Type 2 diabetes	**0.23 (0.04)**	**7.4e^-09^**	1	–
Bone mineral density	-0.02 (0.04)	0.57	**0.09 (0.02)**	**9.8e^-06^**

r_g_, genome-wide genetic correlation coefficient; Pv, p-value for r_g_; SE, standard error; bolded values are statistically significant at p<0.05.

### Candidate shared loci among T2D, BMD and PerioLT from cross-trait MTAG meta-analysis

Given the shared genetic correlation and strong GWAS signals of T2D and BMD, we conducted cross-trait MTAG GWAS meta-analyses between PerioLT/T2D or PerioLT/T2D/BMD to amplify the potentially true but weak genetic signals in the PerioLT GWAS (**Table 2**). The number of genome-wide statistically significant variants from MTAG for the PerioLT GWAS increased from 9 SNPs to 583 when analyzed with T2D, or to 615 SNPs when analyzed together with T2D/BMD. Among these, 548 and 244 SNPs had p_MTAG_<5x10^-8^ across all traits of PerioLT/T2D or PerioLT/T2D/BMD, respectively. We consolidated the PerioLT_MTAG_ signals by multi-clumping the 548 and 244 PerioLT MTAG SNPs into the final 21 SNPs for subsequent colocalization analyses. Of note, out of the 21 SNPs, 11 of them had the original PerioLT GWAS p<0.1 (perio11 in **Tables 2**, **3**).

**Table 2:** MTAG cross-trait significant variants of among PerioLT, T2D, and BMD.

GWAS summary statistics*	N_eff_	Original GWAS # SNPs p<5e^-08^	MTAG PerioLT + T2D # SNPs p_MTAG_<5e^-08^	MTAG PerioLT + T2D + BMD # SNPs p_MTAG_<5e^-08^	Total Independent # SNPs
T2D (Mahajan)	228825	18911	13955	13528	
BMD (Morris)	426824	103257	**–**	79818	
PerioLT (Shungin)	506594	9	583	615	
# SNPs with **p**_MTAG_ **<**5e^-08^ across all traits			548	244	
# SNPs after clumping with p_PerioLT_MTAG_			18	7 (4 overlapping)	21
List of perio11: from 21 SNPs with original GWAS p_PerioLT_ < 0.1			9	5 (3 overlapping)	11

T2D, DIAGRAM type 2 diabetes meta-analysis (23); BMD, GEFOS heel bone mineral density analysis (24.); PerioLT, GWAS of periodontitis/loose teeth from the UKBB/GLIDE (13); N_eff_, effective sample size from the downloaded dataset; Rg, genome-wide genetic correlation coefficient calculated from the LD Score Regression.

**Table 3:** Colocalization posterior probabilities and the original GWAS p-values of 21 SNPs identified from the MTAG analyses.

SNP	Candidacy for common loci	Source of MTAG	Prob H4 PerioLT/T2D	Prob H4 T2D/BMD	Prob H4 PerioLT/BMD	Original GWAS Beta (SE); p_PerioLT_	Original GWAS Beta (SE); p_T2D_	Original GWAS Beta (SE); p_BMD_
Colocalized								
rs17522122	perio11, perio_t2d	MTAG2	0.582	0.008	0.001	0.02 (0.0); 5e^-5^	0.04 (0.01); 4e^-09^	0.0 (0.0); 0.17
Suggestive								
rs3200401	perio11	MTAG2	0.368	0.052	0.345	0.02 (0.01); 8e^-4^	0.06 (0.01); 3e^-14^	-0.03 (0.0); 9e^-23^
rs149290349	perio11	MTAG2&3	0.017	0.977	0.029	-0.02 (0.01); 0.01	-0.13 (0.01); 3e^-24^	-0.03 (0.0); 1e^-15^
rs6711375	perio11	MTAG2	0.008	0.004	0.002	0.02 (0.0); 2e^-4^	0.04 (0.01); 3e^-10^	0.0 (0.0); 0.49
rs1265758*	perio11	MTAG2	0.015	0.004	0.015	-0.01 (0.0); 2e^-3^	-0.05 (0.01); 4e^-13^	0.0 (0.0); 0.03
rs2010390	perio11	MTAG3	0.132	0.015	0.091	0.01 (0.0); 3e^-3^	0.04 (0.01); 4e^-10^	0.03 (0.0); 1e^-40^
rs12255678	perio11	MTAG2&3	0.002	0	0.003	0.01 (0.0); 0.04	-0.15 (0.01); 1e^-85^	-0.02 (0.0); 2e^-13^
rs10770140	perio11	MTAG2	0.114	0.005	0	-0.01 (0.0); 6e^-3^	-0.07 (0.01); 3e^-23^	0.0 (0.0); 0.14
rs8047395	perio11	MTAG2&3	0.002	0.804	0.002	0.01 (0.0); 0.08	0.10 (0.01); 1e^-52^	-0.02 (0.0); 6e^-16^
rs665268	perio11	MTAG3	0.178	0.968	0.081	-0.01 (0.0); 3e^-3^	-0.05 (0.01); 1e^-11^	-0.01 (0.0); 1e^-09^
rs2546494	perio11	MTAG2	0.045	0.009	0	0.01 (0.0); 6e^-4^	0.04 (0.01); 6e^-12^	-0.0 (0.0); 0.12
Additional								
rs4376068	t2d_bmd	MTAG3	0.003	0.955	0.003	0.0 (0.0); 0.45	-0.11 (0.01); 1e^-57^	-0.01 (0.0); 5e^-08^
rs75933965	t2d_bmd	MTAG2	0.002	0	0.003	0.01 (0.01); 0.65	0.23 (0.01); 8e^-75^	-0.0 (0.0); 0.76
rs77464186	t2d_bmd	MTAG2	0.005	0.062	0	0.01 (0.01); 0.26	0.11 (0.01); 2e^-33^	0.01 (0.0); 6e^-3^
rs76895963	t2d_bmd	MTAG2&3	0.008	1	0.008	-0.01 (0.01); 0.64	0.48 (0.03); 5e^-70^	0.08 (0.01); 9e^-25^
Not suggestive								
rs4328980		MTAG2	0.002	0.001	0	-0.0 (0.0); 0.4	-0.08 (0.01); 1e^-35^	0.0 (0.0); 0.41
rs9348440		MTAG2	0.003	0.002	0	0.01 (0.01); 0.21	0.13 (0.01); 3e^-44^	0.0 (0.0); 0.22
rs13266634		MTAG2	0.003	0.003	0	-0.0 (0.0); 0.49	-0.11 (0.01); 1e^-54^	0.0 (0.0); 0.29
rs7020996		MTAG2	0.003	0.011	0	0.0 (0.0); 0.8	-0.15 (0.01); 4e^-52^	-0.0 (0.0); 0.12
rs11257655		MTAG2	0.009	0.008	0	0.01 (0.0); 0.11	0.09 (0.01); 4e^-32^	0.0 (0.0); 0.08
rs61875362		MTAG2	0.003	0.002	0	-0.0 (0.0); 0.4	-0.09 (0.01); 2e^-41^	-0.0 (0.0); 1

Prob, probability; H4, hypothesis of sharing 1 lead SNP; T2D, DIAGRAM type 2 diabetes meta-analysis (23); MTAG2&3, from both MTAG2 and MTAG3, with results of MTAG3 shown here; BMD, GEFOS heel bone mineral density analysis (24); PerioBL, GWAS of periodontitis/loose teeth from the UKBB/GLIDE (13); MTAG, multi-trait analysis of GWAS; MTAG2, MTAG of PerioLT and T2D; MTAG3, MTAG of PerioLT, BMD, and T2D; *, rs1265758 was not available in the WGHS data.

Candidacy for common loci annotation: perio_t2d, SNP identified with H4 probability of T2D/PerioLT colocalization >0.5; perio11, SNP identified from the MTAG analysis and with the original GWAS p_perioLT_<0.1: t2d_bmd, additional SNP identified with H4 probability of T2D/BMD colocalization >0.7 or higher H3 probability of 2 different lead SNPs in T2D/BMD.

### Pairwise colocalization analyses of candidate SNP associations between PerioLT/T2D, T2D/BMD and BMD/PerioLT

At the candidate loci, we evaluated colocalization of genetic signals for pairs of the examined phenotypes. **Table 3** shows the posterior probabilities of the hypothesis H4, which supports sharing of a causal variant between the phenotypes. **Tables S1–S3** show the posterior probabilities of all alternative hypotheses (H1-H4). Of the 21 variants identified by MTAG (**Table 2**), one SNP (rs17522122) colocalized for PerioLT/T2D (H4 = 0.582), while none of the other colocalization alternatives for this variant involving PerioLT was significant (H1, H3 <0.05). The colocalization evidence for shared association at a second variant, rs3200401, was marginal for both PerioLT/T2D and PerioLT/BMD (H4 = 0.37 and 0.35, respectively). At an additional locus indexed by rs6711375, colocalization suggests there are independent associations for PerioLT and for T2D (**Table S1**; H3 = 0.905, H4 = 0.008). No additional candidate was supported for association with PerioLT by colocalization analysis, despite having PerioLT p_MTAG_<5x10^-8^. However, there was support for shared associations at between T2D and BMD at five loci (SNP=rs149290349, H4 = 0.977; rs8047395, 0.804; rs665268, 0.968; rs4376068, 0.955; rs76895963, 1.00), and support for independent associations for T2D and BMD at four loci (SNP=rs3200401, H3 = 0.948; rs1265758, 0.645; rs2010390, 0.985; rs75933965, 1.00), the first also a PerioLT candidate (above). We also retained rs77464186 for further analysis based on the elevated although not significant H3 probabilities (0.163). Among the candidate SNPs, those that showed no evidence for association (P>0.1) in the original PerioLT GWAS are designated in the tables as “t2d_bmd” if there was colocalization between T2D and BMD.

### Description of the remaining candidate shared loci and their MTAG results

**Table 4** provides details of gene symbols, chromosome and position, allele frequency, type of variant, as well as the MTAG summary statistics of the 15 selected SNPs. The colocalized SNP between PerioLT and T2D, rs17522122, maps to the 3’-UTR of *AKAP6*. The A-kinase anchor proteins are a group of proteins highly expressed in various brain regions, cardiac and skeletal muscle and tongue, with biological functions involved in anchoring protein kinase A to the nuclear membrane or sarcoplasmic reticulum. The marginally colocalizing SNP for PerioLT, rs3200401, maps to a non-coding RNA near *MALAT1*, *MASCRNA* and *TALAM1*. Seven SNPs were derived from the three-trait MTAG analysis and had the following genomic contexts: rs149290349 (a missense variant of *ZFP36L2*), rs2010390 (an uncharacterized non-coding RNA), rs12255678 (an intron variant of *TCF7L2*), rs8047395 (an intron variant of *FTO*), rs665268 (a missense variant of *MLX*), rs4376068 (an intron variant of *IGF2BP2*), and rs76895963 (an intron variant of *CCND1, CCND2-AS1*). Among these, 4 SNPs (rs149290349, rs12255678, rs8047395, and rs76895963) were also found to be significant in the PerioLT/T2D two-trait MTAG analysis.

**Table 4:** MTAG association statistics for 15 selected variants.

SNP	Chr : Pos	A1	A2	Freq	Source of MTAG	Type	Candidate Genes	Beta (SE); p_PerioLT_MTAG_	Beta (SE); p_T2D_MTAG_	Beta (SE); p_BMD_MTAG_	
Colocalized											
rs17522122	14:33302882	T	G	0.48	MTAG2	3’UTR	*AKAP6*	0.02 (0.0); 1e^-08^	0.04 (0.01); 2e^-09^		
Suggestive											
rs3200401	11:65271832	T	C	0.20	MTAG2	ncRNA	*MALAT1, MASCRNA*, *TALAM1*	0.03 (0.0); 9e^-09^	0.06 (0.01); 1e^-13^		
rs149290349	2:43451957	A	G	0.07	MTAG2&3	missense	*ZFP36L2*	-0.04 (0.01); 3e^-10^	-0.13 (0.01); 3e^-21^	-0.03 (0.0); 1e^-12^	
rs6711375	2:161090873	A	G	0.68	MTAG2	SNV	*–*	0.02 (0.0); 3e^-08^	0.05 (0.01); 2e^-10^		
rs1265758	6:32323529	A	G	0.42	MTAG2	intron	*TSBP1, TSBP1-AS1*	-0.02 (0.0); 3e^-08^	-0.05 (0.01); 9e^-13^	
rs2010390	8:9047178	A	G	0.30	MTAG3	intron	*LOC101929128*	0.02 (0.0); 2e^-08^	0.04 (0.01); 9e^-09^	0.03 (0.0); 3e^-44^	
rs12255678	10:114729482	T	G	0.75	MTAG2&3	intron	*TCF7L2*	0.02 (0.0); 6e^-11^	-0.15 (0.01); 8e^-81^	-0.02 (0.0); 6e^-10^	
rs10770140	11:2193597	T	C	0.61	MTAG2	5’ UTR	*TH, MIR4686*	-0.02 (0.0); 8e^-10^	-0.07 (0.01); 1e^-22^		
rs8047395	16:53798523	A	G	0.51	MTAG2&3	intron	*FTO*	0.03 (0.0); 2e^-12^	0.10 (0.01); 2e^-51^	-0.02 (0.0); 1e^-17^	
rs665268	17:40722029	A	G	0.72	MTAG3	missense	*MLX*	-0.02 (0.0); 5e^-08^	-0.05 (0.01); 4e^-11^	-0.01 (0.0); 3e^-08^	
rs2546494	17:46959525	A	G	0.51	MTAG2	intron	*LOC105371814*	0.02 (0.0); 3e^-08^	0.04 (0.01); 8e^-12^		
Additional											
rs4376068	3:185497635	A	C	0.69	MTAG3	intron	*IGF2BP2*	-0.02 (0.0); 2e^-09^	-0.11 (0.01); 2e^-52^	-0.01 (0.0); 6e^-09^	
rs75933965	10:114749421	A	G	0.07	MTAG2	intron	*TCF7L2*	0.05 (0.01); 6e^-13^	0.23 (0.01); 7e^-65^		
rs77464186	11:72460398	A	C	0.84	MTAG2	intron	*ARAP1*	0.03 (0.01); 8e^-09^	0.11 (0.01); 1e^-33^		
rs76895963	12:4384844	T	G	0.98	MTAG2&3	intron	*CCND1, CCND2-AS1*	0.08 (0.01); 2e^-09^	0.48 (0.03); 1e^-62^	0.08 (0.01); 7e^-19^	

MTAG, multi-trait analysis of GWAS; MTAG2, MTAG of PerioLT and T2D; MTAG3, MTAG of PerioLT, BMD, and T2D; MTAG2&3, from both MTAG2 and MTAG3, with results of MTAG3 shown here; Chr : Pos, chromosome and position (GRCh37/hg19); Freq, allele frequency of A1; SE, standard error; p_PerioLT_MTAG_, MTAG calculated p-values of the periodontitis/loose teeth from the UKBB/GLIDE (13); p_T2D_MTAG_, MTAG calculated p-values of the type 2 diabetes (23); p_BMD_MTAG_, MTAG calculated p-values of the GEFOS bone mineral density (24).

### Characteristics of the Women's Genome Health Study participants with updated oral health measures

We provide characteristics of the WGHS participants who had verified European ancestry, had completed the oral health questionnaire (OHQ), and had information on their T2D status throughout the follow-up period in **Supplementary Table S4**. Distribution and risk factors for periodontal status from responses to OHQ, such as age at the time of the OHQ, baseline educational levels, smoking status, BMI, hypertension, hypercholesterolemia and updated osteoporosis status as are provided based on the confirmed T2D status. Women with T2D were more likely to self-report having fair/poor oral health, less likely to visit a dentist within the past year, less likely to have dental prophylaxis at least once per year, and more likely to floss two times or less per week. Genotype distributions of the shared candidate loci among the WGHS participants are provided in **Supplementary Table S5**. We were able to retrieve clinical dental records from a subset of women (**Supplementary Table S6**). Women who self-reported having fair/poor oral health, bone loss around teeth, or those with less dental prophylaxis (<1 per year), had fewer remaining natural teeth. Thus, in the WGHS, responses to the OHQ demonstrated associations with worse PD status consistent with previous validation studies.

### Genetic associations of the candidate shared PerioLT/T2D variants with responses to the oral health questions

**Table 5** presents the genetic associations for 14 of the 15 selected variants with responses of OHQs among the WGHS participants. Data on one variant, rs1265758, were unavailable in the WGHS. The influence of each genetic variant on oral health measures was explored in multiple logistic regression models (**Table 5** legend). Rs17522122, the top candidate variant that colocalized between PerioLT and T2D, was found to be inversely associated with less frequent dental flossing [OR(95%CI)= 0.92 (0.87-0.98), p=0.007]. Additional variants were found to be associated with the women’s last visit to the dental office [rs77464186, OR(95%CI)= 0.82 (0.75-0.91), p=0.0002], frequency of dental prophylaxis [rs67111375, 0.91 (0.83-0.99), p=0.03; rs77464186, 0.80 (0.72-0.89), p=3.8e^-05^], frequency of dental flossing [rs75933965, 1.17 (1.04-1.31), p=0.008], and a history of bone loss around teeth [rs8047395, 1.09 (1.03-1.15), p=0.005].

**Table 5:** Oral health question (OHQs) with at least one significant genetic association.

	Dental Visit > 1 year ago		Prophylaxis < 1 per year		Floss <= 2 per week		Bone loss around teeth	
	OR (95%CI)	p	OR (95%CI)	p	OR (95%CI)	p	OR (95%CI)	p
Colocalized								
rs17522122_t	0.96 (0.89-1.04)	0.32	1 (0.92-1.09)	0.98	0.92 (0.87-0.98)	0.007	1.05 (0.99-1.11)	0.13
Suggestive								
rs3200401_t	1.03 (0.94-1.14)	0.49	1.07 (0.97-1.18)	0.19	1.07 (0.99-1.15)	0.09	0.98 (0.91-1.06)	0.68
rs149290349_a	0.87 (0.75-1.01)	0.08	0.9 (0.77-1.05)	0.20	1.04 (0.93-1.16)	0.47	0.96 (0.86-1.08)	0.50
rs6711375_a	0.94 (0.87-1.02)	0.15	0.91 (0.83-0.99)	0.03	1.02 (0.95-1.08)	0.62	1 (0.94-1.07)	0.88
rs2010390_a	1.01 (0.92-1.11)	0.84	1.07 (0.97-1.18)	0.19	0.97 (0.91-1.05)	0.48	1.04 (0.97-1.11)	0.31
rs12255678_t	1.02 (0.93-1.12)	0.64	1.02 (0.93-1.13)	0.61	1.03 (0.96-1.1)	0.43	0.99 (0.92-1.06)	0.77
rs10770140_t	1.03 (0.95-1.11)	0.52	1.08 (1-1.18)	0.06	1.02 (0.96-1.08)	0.54	0.97 (0.91-1.03)	0.25
rs8047395_a	1.04 (0.96-1.12)	0.35	1.02 (0.94-1.11)	0.59	0.98 (0.92-1.04)	0.55	1.09 (1.03-1.15)	0.005
rs665268_a	0.98 (0.9-1.07)	0.65	0.95 (0.86-1.04)	0.22	0.95 (0.89-1.01)	0.11	1.02 (0.95-1.08)	0.66
rs2546494_a	0.99 (0.91-1.06)	0.71	0.97 (0.9-1.05)	0.50	1.02 (0.96-1.08)	0.56	1 (0.94-1.06)	0.98
Additional								
rs4376068_a	0.96 (0.88-1.04)	0.28	0.99 (0.91-1.08)	0.84	0.97 (0.91-1.03)	0.35	1.02 (0.96-1.09)	0.53
rs75933965_a	1.02 (0.87-1.18)	0.83	0.97 (0.82-1.14)	0.69	1.17 (1.04-1.31)	0.008	0.97 (0.86-1.09)	0.63
rs77464186_a	0.82 (0.75-0.91)	0.0002	0.8 (0.72-0.89)	3.8e^-05^	0.96 (0.88-1.04)	0.29	0.99 (0.92-1.08)	0.90
rs76895963_t	0.8 (0.61-1.08)	0.14	0.91 (0.67-1.28)	0.58	0.86 (0.69-1.1)	0.22	1.09 (0.85-1.4)	0.51

The associations to different oral health question responses were assessed with multivariate logistic regression using the genetic variables as the independent variables, adjusting for age at the time of OHQ responses, baseline information smoking (3 groups), and 10 genomic eigenvectors.

OR, odds ratio; CI, confidence interval.

By further controlling for other traditional risk factors such as baseline BMI, education, hypertension and hypercholesterolemia, as well as the type 2 diabetes and osteoporosis status updated over follow-up, results of the genetic associations confirmed those shown in **Table 4**, though slightly attenuated (**Supplementary Table S7**). This observation suggests that the genetic effects are not functioning through these clinical conditions.

Lastly, we did not find significant genetic associations for these 14 variants with self-reported poor/fair oral health, a history of PD diagnosis, or a history of scaling and root planing (**Supplementary Table S8**).

### Pleiotropy at candidate loci and tissues specific expression quantitative trait loci

We provide additional annotations for two variants associated with less frequent dental flossing (rs17522122 and rs75933965), two associated with less frequent dental prophylaxis (rs6711375, rs77464186), and the variant rs8037495 associated with bone loss around teeth (**Supplementary Table S9**). Multiple diverse phenome-wide association studies (PheWAS) results were identified for all five variants, implying a high level of pleiotropy. For example, GWAS associations with rs17522122 were enriched in phenotypes related to body fat. Rs6711375 and rs8047395 were identified with pleiotropic effects related to a variety of immune cell (leukocyte, lymphocyte, neutrophil and reticulocytes). The most pleiotropic associations highlighted were diabetes, body fat, body mass and body measures, and metabolic related traits. The variants associated with less frequent dental prophylaxis were also linked to expression quantitative trait loci, i.e., eQTLs, of several genes. Lastly, blood pressure, cancer, cognitive function or physical activities GWAS results were previously reported with rs17522122. The *FTO* variant (rs8047395) was also mapped with tumors of central nervous system or other cancers, as well as bone mineral density.

## Discussion

A possible explanation for relative lack of significant loci in the former UKBB/GLIDE PerioLT GWAS meta-analysis despite its large sample size may have been the potential heterogeneity in phenotype ascertainment across the contributing studies. To overcome this challenge, we boosted the PerioLT genetic signal with cross-trait MTAG meta-analysis, thereby identifying 21 genome-wide significant associations for PerioLT (after clumping), among which one was also nominally significant in the original GWAS and colocalized with the genetic signal for T2D (rs17522122). Testing for association with OHQs among the WGHS women, including adjustment for traditional risk factors and updated histories of diabetes and osteoporosis, supported some associations with less frequent dental care/prophylaxis (rs77464186) or less frequent flossing (rs17522122), or bone loss around teeth (rs8047359) despite the relatively small size of the sample. Rs6711375, which tagged a locus with potentially distinct signals for PerioLT and T2D (H3 = 0.91) and was marginally supported by association with less dental prophylaxis via OHQ, may have had a strong PerioLT MTAG signal due to inflation *via* residual LD to the strong T2D signal. Similarly, significantly replicating associations with dental phenotypes that did not colocalize with PerioLT are likely due to the highly pleiotropic nature of these candidate loci and their particularly significant associations with T2D and/or BMD, which may have inflated MTAG signals for PerioLT (26). For example, rs12255678 near *TCF7L2* and rs8047395 near *FTO* are the strongest associations in the genome for T2D and BMD, respectively. Therefore, we acknowledged such limitation from the MTAG results that these suggested shared PD candidate loci may only reflect very strong T2D or BMD associations.

That loci showing pleiotropy across a range of immune cell phenotypes and that T2D and BMD are associated with periodontal measures is consistent with current thinking about the origins of periodontal disease. Williams and colleagues report that cellular transcriptomic landscape of patients with periodontitis involved enhanced neutrophil and leukocyte infiltration due to the exaggerated stromal cell responsiveness (2). Meanwhile, bone changes are known among diabetic patients, perhaps with partial microvascular etiology (12), and mouse models link obesity and its metabolic dysregulation-associated inflammation to the size of preosteoclasts (myeloid-derived suppressor cells) populations (34). Both the genetic correlation of T2D/BMD and the colocalization of signals at some candidate loci are consistent with previously reported connections between these phenotypes from Mendelian randomization studies (35). Our result of lack of genetic correlation between PerioLT and BMD does not support a genetic relationship for the reported epidemiological associations between PD and osteoporosis (6, 33). We further suggest that the opposite direction of genetic correlation between PerioLT/BMD and T2D/BMD might imply different pathophysiology for alveolar bone loss than those measurements identified under the osteoporotic conditions.

A strength of our approach is the large sample size based on the consortium GWAS datasets as well as the WGHS/WHS genetic data for validations. Additionally, obtaining the validated CDC-AAP OHQs in the WGHS/WHS likely reduced heterogeneity in the periodontal phenotype that may have limited genetic signal in the original PerioLT GWAS. We do acknowledge that even as such OHQs have good sensitivity and specificity for periodontal disease surveillance (18), they may nevertheless be limited by the nature of self-reporting. Nevertheless, among the limited women’s dental records retrieved in the WGHS/WHS, participants having less frequent dental prophylaxis, bone loss around teeth, or who self-reported fair/poor oral health had significantly fewer remaining teeth. Thus, the identified genetic associations between the reported shared PD/T2D candidate loci with responses to OHQs in the WGHS may be interpreted as genetic liability to surrogates for PD. Our integrated approach supports deployment of validated OHQs in future genomic studies so that important oral health information can be captured.

In summary, by exploring genetic analyses using GWAS summary statistics of PerioLT, T2D and BMD, we were able to identify one new candidate locus for PerioLT and several additional new suggestive loci. Among the WGHS women, significant genetic associations of these candidate variants with self-reported oral health measures remained even after accounting for other risk factors and the women’s osteoporosis and diabetes status. Importantly, our observations may bear on significant association of periodontal disease or having a less than functional dentition with many systemic comorbidities such as T2D, cardiovascular disease (36), bone mineral density and hip fracture (37), as well as all-cause or disease-specific mortality (38). Future research must explore how the new loci are linked to underlying pathophysiology of periodontal diseases and its comorbidities.

## Data availability statement

The original contributions presented in the study are included in the article/**Supplementary Material**. Further inquiries can be directed to the corresponding author.

## Ethics statement

The studies involving human participants were reviewed and approved by the Institutional Review Board of Brigham and Women’s Hospital, Boston Massachusetts. The patients/participants provided their written informed consent to participate in this study.

## Author contributions

Y-HY contributed to conception, design, data collection, data analysis, data interpretation and drafted the manuscript. JB and DC contributed to conception, design, data collection, data interpretation and critically revised the manuscript. BS and PR contributed to conception, design and critically revised the manuscript. All authors contributed to the article and approved the submitted version.
