# Supplementary material for: Candidate loci shared among periodontal disease, diabetes and bone density
Source: Front Endocrinol (Lausanne). 2023 Jan 27;13:1016373. doi: 10.3389/fendo.2022.1016373 (PMC9911896; doi:10.3389/fendo.2022.1016373)
Supplement: Supplementary file 1 [file DataSheet_1.docx]

**Supplementary Tables**

**Supplementary Table S1**

Colocalization analysis between GWASs of PerioLT and T2D

**Supplementary Table S2**

Colocalization analysis between GWASs of T2D and BMD

**Supplementary Table S3**

Colocalization analysis between GWASs of PerioLT and BMD

**Supplementary Table S4**

Characteristics of the WGHS study participants based on the confirmed type 2 diabetes (T2D)

**Supplementary Table S5**

Distribution of genetic information of the identified shared loci among the WGHS women based on the confirmed type 2 diabetes (T2D)

**Supplementary Table S6**

Clinical dental characteristics related to responses of OHQs from a limited subset of WGHS women with retrieved dental records

**Supplementary Table S7**

Significant genetic associations with responses to oral health questions (OHQs) adjusting for more traditional risk factors

**Supplementary Table S8**

Non-significant genetic associations with responses to oral health questions (OHQs)

**Supplementary Table S9**

Summary of information from PheWAS, GWAS catalog and eQTLs for selected candidate loci

Suppl. Table S1 Colocalization analysis between GWASs of PerioLT and T2D

| **Loci/SNP** | **Candidacy**  **for**  **common loci** | **Chr:Pos** | | **Source of MTAG** | **Prob H0**  **No causal SNP in both traits** | **Prob H1**  **Causal SNP**  **in PerioLT** | **Prob H2**  **Causal SNP**  **in T2D** | **Prob H3**  **2 diff SNPs**  **in PerioLT/T2D** | **Prob H4**  **Share 1 SNP in PerioLT/T2D** |
| --- | --- | --- | --- | --- | --- | --- | --- | --- | --- |
| ***Colocalized*** |  |  | |  |  |  |  |  |  |
| rs17522122 | perio11, perio_t2d | 14:33302882 | | MTAG2 | 0 | 0 | 0.407 | 0.011 | 0.582 |
| ***Suggestive*** |  |  | |  |  |  |  |  |  |
| rs3200401 | perio11 | 11:65271832 | | MTAG2 | 0 | 0 | 0.593 | 0.039 | 0.368 |
| rs149290349 | perio11 | 2:43451957 | | MTAG2&3 | 0 | 0 | 0.967 | 0.016 | 0.017 |
| rs6711375 | perio11 | 2:161090873 | | MTAG2 | 0 | 0 | 0.087 | 0.905 | 0.008 |
| rs1265758* | perio11 | 6:32323529 | | MTAG2 | 0 | 0 | 0.967 | 0.018 | 0.015 |
| rs2010390 | perio11 | 8:9047178 | | MTAG3 | 0 | 0 | 0.865 | 0.003 | 0.132 |
| rs12255678 | perio11 | 10:114729482 | | MTAG2&3 | 0 | 0 | 0.991 | 0.007 | 0.002 |
| rs10770140 | perio11 | 11:2193597 | | MTAG2 | 0 | 0 | 0.879 | 0.006 | 0.114 |
| rs8047395 | perio11 | 16:53798523 | | MTAG2&3 | 0 | 0 | 0.987 | 0.01 | 0.002 |
| rs665268 | perio11 | 17:40722029 | | MTAG3 | 0 | 0 | 0.798 | 0.024 | 0.178 |
| rs2546494 | perio11 | 17:46959525 | | MTAG2 | 0 | 0 | 0.914 | 0.041 | 0.045 |
| ***Additional*** |  |  | |  |  |  |  |  |  |
| rs4376068 | t2d_bmd | 3:185497635 | | MTAG3 | 0 | 0 | 0.991 | 0.006 | 0.003 |
| rs75933965 | t2d_bmd | 10:114749421 | | MTAG2 | 0 | 0 | 0.991 | 0.007 | 0.002 |
| rs77464186 | t2d_bmd | 11:72460398 | | MTAG2 | 0 | 0 | 0.992 | 0.003 | 0.005 |
| rs76895963 | t2d_bmd | 12:4384844 | | MTAG2&3 | 0 | 0 | 0.992 | 0.001 | 0.008 |
| ***Not suggestive*** | | |  |  |  |  |  |  |  |
| rs4328980 |  | 4:6281756 | | MTAG2 | 0 | 0 | 0.997 | 0.001 | 0.002 |
| rs9348440 |  | 6:20641336 | | MTAG2 | 0 | 0 | 0.985 | 0.012 | 0.003 |
| rs13266634 |  | 8:118184783 | | MTAG2 | 0 | 0 | 0.967 | 0.03 | 0.003 |
| rs7020996 |  | 9:22129579 | | MTAG2 | 0 | 0 | 0.992 | 0.005 | 0.003 |
| rs11257655 |  | 10:12307894 | | MTAG2 | 0 | 0 | 0.991 | 0.001 | 0.009 |
| rs61875362 |  | 10:94208397 | | MTAG2 | 0 | 0 | 0.991 | 0.006 | 0.003 |

**Abbreviations:** Prob, probability; H, hypothesis; T2D, DIAGRAM type 2 diabetes meta-analysis (Mahajan et al.); BMD, GEFOS heel bone mineral density analysis (Morris et al.); PerioBL, GWAS of periodontitis/loose teeth from the UKB/GLIDE (Shungin et al.); MTAG, multi-trait analysis of GWAS; MTAG2, MTAG of PerioLT and T2D; MTAG3, MTAG of PerioLT, BMD, and T2D; MTAG2&3, from both MTAG2 and MTAG3; *, rs1265758 was not available in the WGHS data.

Candidacy for common loci annotation: perio_t2d, SNP identified with H4 probability of T2D/PerioLT colocalization >0.5; perio11, SNP identified from the MTAG analysis and with the original GWAS p_perioLT_<0.1: t2d_bmd, additional SNP identified with H4 probability of T2D/BMD colocalization >0.7 or higher H3 probability of 2 different lead SNPs in T2D/BMD.

Suppl. Table S2 Colocalization analysis between GWASs of T2D and BMD

| **Loci/SNP** | **Candidacy**  **for**  **common loci** | **Chr:Pos** | | **Source of**  **MTAG** | **Prob H0**  **No causal SNP in both traits** | | **Prob H1**  **Causal SNP**  **in T2D** | | **Prob H2**  **Causal SNP**  **in BMD** | | **Prob H3**  **2 diff SNPs**  **in T2D/BMD** | | **Prob H4**  **Share 1 SNP in T2D/BMD** | |
| --- | --- | --- | --- | --- | --- | --- | --- | --- | --- | --- | --- | --- | --- | --- |
| ***Colocalized*** |  |  | |  |  | |  | |  | |  | |  | |
| rs17522122 | perio11, perio_t2d | 14:33302882 | | MTAG2 | 0.001 | | 0.929 | | 0 | | 0.061 | | 0.008 | |
| ***Suggestive*** |  |  | |  |  | |  | |  | |  | |  | |
| rs3200401 | perio11 | 11:65271832 | | MTAG2 | 0 | | 0 | | 0 | | 0.948 | | 0.052 | |
| rs149290349 | perio11 | 2:43451957 | | MTAG2&3 | 0 | | 0 | | 0 | | 0.023 | | 0.977 | |
| rs6711375 | perio11 | 2:161090873 | | MTAG2 | 0 | | 0.956 | | 0 | | 0.04 | | 0.004 | |
| rs1265758* | perio11 | 6:32323529 | | MTAG2 | 0 | | 0.351 | | 0 | | 0.645 | | 0.004 | |
| rs2010390 | perio11 | 8:9047178 | | MTAG3 | 0 | | 0 | | 0 | | 0.985 | | 0.015 | |
| rs12255678 | perio11 | 10:114729482 | | MTAG2&3 | 0 | | 0 | | 0 | | 1 | | 0 | |
| rs10770140 | perio11 | 11:2193597 | | MTAG2 | 0 | | 0.977 | | 0 | | 0.018 | | 0.005 | |
| rs8047395 | perio11 | 16:53798523 | | MTAG2&3 | 0 | | 0 | | 0 | | 0.196 | | 0.804 | |
| rs665268 | perio11 | 17:40722029 | | MTAG3 | 0 | | 0 | | 0 | | 0.032 | | 0.968 | |
| rs2546494 | perio11 | 17:46959525 | | MTAG2 | 0 | | 0.977 | | 0 | | 0.015 | | 0.009 | |
| ***Additional*** |  |  | |  |  | |  | |  | |  | |  | |
| rs4376068 | t2d_bmd | 3:185497635 | | MTAG3 | 0 | | 0 | | 0 | | 0.044 | | 0.955 | |
| rs75933965 | t2d_bmd | 10:114749421 | | MTAG2 | 0 | | 0 | | 0 | | 1 | | 0 | |
| rs77464186 | t2d_bmd | 11:72460398 | | MTAG2 | 0 | | 0.775 | | 0 | | 0.163 | | 0.062 | |
| rs76895963 | t2d_bmd | 12:4384844 | | MTAG2&3 | 0 | | 0 | | 0 | | 0 | | 1 | |
| ***Not suggestive*** | | |  |  |  |  | |  | |  | |  | |  |
| rs4328980 |  | 4:6281756 | | MTAG2 | 0 | | 0.992 | | 0 | | 0.006 | | 0.001 | |
| rs9348440 |  | 6:20641336 | | MTAG2 | 0 | | 0.985 | | 0 | | 0.013 | | 0.002 | |
| rs13266634 |  | 8:118184783 | | MTAG2 | 0 | | 0.99 | | 0 | | 0.007 | | 0.003 | |
| rs7020996 |  | 9:22129579 | | MTAG2 | 0 | | 0.982 | | 0 | | 0.007 | | 0.011 | |
| rs11257655 |  | 10:12307894 | | MTAG2 | 0 | | 0.979 | | 0 | | 0.012 | | 0.008 | |
| rs61875362 |  | 10:94208397 | | MTAG2 | 0 | | 0.987 | | 0 | | 0.012 | | 0.002 | |

**Abbreviations:** Prob, probability; H, hypothesis; T2D, DIAGRAM type 2 diabetes meta-analysis (Mahajan et al.); BMD, GEFOS heel bone mineral density analysis (Morris et al.); PerioBL, GWAS of periodontitis/loose teeth from the UKB/GLIDE (Shungin et al.); MTAG, multi-trait analysis of GWAS; MTAG2, MTAG of PerioLT and T2D; MTAG3, MTAG of PerioLT, BMD, and T2D; MTAG2&3, from both MTAG2 and MTAG3; *, rs1265758 was not available in the WGHS data.

Candidacy for common loci annotation: perio_t2d, SNP identified with H4 probability of T2D/PerioLT colocalization >0.5; perio11, SNP identified from the MTAG analysis and with the original GWAS p_perioLT_<0.1: t2d_bmd, additional SNP identified with H4 probability of T2D/BMD colocalization >0.7 or higher H3 probability of 2 different lead SNPs in T2D/BMD.

Suppl. Table S3 Colocalization analysis between GWASs of PerioLT and BMD

| **Loci/SNP** | **Candidacy**  **for**  **common loci** | **Chr:Pos** | | | **Source of MTAG** | **Prob H0**  **No causal SNP in both traits** | | **Prob H1**  **Causal SNP**  **in PerioLT** | | **Prob H2**  **Causal SNP**  **in BMD** | | **Prob H3**  **2 diff SNPs**  **in PerioLT/BMD** | | **Prob H4**  **Share 1 SNP in PerioLT/BMD** | |
| --- | --- | --- | --- | --- | --- | --- | --- | --- | --- | --- | --- | --- | --- | --- | --- |
| ***Colocalized*** |  |  | | |  |  | |  | |  | |  | |  | |
| rs17522122 | perio11, perio_t2d | | 14:33302882 | | MTAG2 | 0.913 | | 0.025 | | 0.06 | | 0.002 | | 0.001 | |
| ***Suggestive*** |  | |  | |  |  | |  | |  | |  | |  | |
| rs3200401 | perio11 | | 11:65271832 | | MTAG2 | 0 | | 0 | | 0.615 | | 0.04 | | 0.345 | |
| rs149290349 | perio11 | | 2:43451957 | | MTAG2&3 | 0 | | 0 | | 0.963 | | 0.009 | | 0.029 | |
| rs6711375 | perio11 | | 2:161090873 | | MTAG2 | 0.092 | | 0.868 | | 0.004 | | 0.034 | | 0.002 | |
| rs1265758* | perio11 | | 6:32323529 | | MTAG2 | 0.607 | | 0.01 | | 0.361 | | 0.006 | | 0.015 | |
| rs2010390 | perio11 | | 8:9047178 | | MTAG3 | 0 | | 0 | | 0.905 | | 0.003 | | 0.091 | |
| rs12255678 | perio11 | | 10:114729482 | | MTAG2&3 | 0 | | 0 | | 0.992 | | 0.005 | | 0.003 | |
| rs10770140 | perio11 | | 11:2193597 | | MTAG2 | 0.992 | | 0.008 | | 0.001 | | 0 | | 0 | |
| rs8047395 | perio11 | | 16:53798523 | | MTAG2&3 | 0 | | 0 | | 0.992 | | 0.006 | | 0.002 | |
| rs665268 | perio11 | | 17:40722029 | | MTAG3 | 0 | | 0 | | 0.895 | | 0.025 | | 0.081 | |
| rs2546494 | perio11 | | 17:46959525 | | MTAG2 | 0.949 | | 0.038 | | 0.012 | | 0 | | 0 | |
| ***Additional*** |  | |  | |  |  | |  | |  | |  | |  | |
| rs4376068 | t2d_bmd | | 3:185497635 | | MTAG3 | 0.004 | | 0 | | 0.989 | | 0.004 | | 0.003 | |
| rs75933965 | t2d_bmd | | 10:114749421 | | MTAG2 | 0 | | 0 | | 0.992 | | 0.005 | | 0.003 | |
| rs77464186 | t2d_bmd | | 11:72460398 | | MTAG2 | 0.962 | | 0.003 | | 0.035 | | 0 | | 0 | |
| rs76895963 | t2d_bmd | | 12:4384844 | | MTAG2&3 | 0 | | 0 | | 0.992 | | 0.001 | | 0.008 | |
| ***Not suggestive*** | | | |  |  |  |  | |  | |  | |  | |  |
| rs4328980 |  | | 4:6281756 | | MTAG2 | 0.999 | | 0.001 | | 0.001 | | 0 | | 0 | |
| rs9348440 |  | | 6:20641336 | | MTAG2 | 0.979 | | 0.01 | | 0.011 | | 0 | | 0 | |
| rs13266634 |  | | 8:118184783 | | MTAG2 | 0.969 | | 0.026 | | 0.004 | | 0 | | 0 | |
| rs7020996 |  | | 9:22129579 | | MTAG2 | 0.992 | | 0.003 | | 0.004 | | 0 | | 0 | |
| rs11257655 |  | | 10:12307894 | | MTAG2 | 0.999 | | 0.001 | | 0.001 | | 0 | | 0 | |
| rs61875362 |  | | 10:94208397 | | MTAG2 | 0.986 | | 0.004 | | 0.01 | | 0 | | 0 | |

**Abbreviations:** Prob, probability; H, hypothesis; T2D, DIAGRAM type 2 diabetes meta-analysis (Mahajan et al.); BMD, GEFOS heel bone mineral density analysis (Morris et al.); PerioBL, GWAS of periodontitis/loose teeth from the UKB/GLIDE (Shungin et al.); MTAG, multi-trait analysis of GWAS; MTAG2, MTAG of PerioLT and T2D; MTAG3, MTAG of PerioLT, BMD, and T2D; MTAG2&3, from both MTAG2 and MTAG3; *, rs1265758 was not available in the WGHS data.

Candidacy for common loci annotation: perio_t2d, SNP identified with H4 probability of T2D/PerioLT colocalization >0.5; perio11, SNP identified from the MTAG analysis and with the original GWAS p_perioLT_<0.1: t2d_bmd, additional SNP identified with H4 probability of T2D/BMD colocalization >0.7 or higher H3 probability of 2 different lead SNPs in T2D/BMD.

Suppl. Table S4 Characteristics of the WGHS study participants based on the confirmed type 2 diabetes (T2D)

|  | | Total | | No T2D | | | Confirmed T2D | ***P*** ^b^ | |
| --- | --- | --- | --- | --- | --- | --- | --- | --- | --- |
| N | | 14663 | | 13271 (91) | | | 1392 (9) |  | |
| Age in 2018 ^a^ | | 77.0 (5.6) | | 77.01(5.5) | | | 77.35(5.7) | 0.03 | |
| ***Baseline Information*** |  | | | |  | | |  | |
| Education | |  | |  | | |  | <.001 | |
| < Bachelor’s degree | | 14615 (96) | | 6767 (51.0) | | | 842 (60.5) |  | |
| > Bachelor’s degree | | 225 (1.5) | | 6504 (49.0) | | | 550 (39.5) |  | |
| Smoking | |  | |  | | |  | 0.19 | |
| Current | | 1286 (8.7) | | 1130 (8.5) | | | 139 (10.0) |  | |
| Past | | 5591 (37.6) | | 4995 (37.7) | | | 515 (37.0) |  | |
| Never | | 7979 (53.7) | | 7138 (53.8) | | | 738 (53.0) |  | |
| BMI | | 25.60 (4.7) | | 25.11 (4.3) | | | 30.28 (5.8) | <.001 | |
| Hypertension | | 2873 (19.6) | | 2309 (17.4) | | | 564 (40.6) | <.001 | |
| Hypercholesterolemia | | 3859(26.3) | | 3316 (25.0) | | | 543 (39.1) | <.001 | |
| Osteoporosis (2009) | | 2266 (15.5) | | 2107 (18.5) | | | 159 (14.4) | <.001 | |
| ***Oral health questions (2018)*** | | |  | | |  | | |  |
| Prophylaxis < once per year | | 1314 (9.0) | | 1112 (8.5) | | | 202 (15.1) | <.001 | |
| Last dental visit (> 1 year ago) | | 1501 (10.2) | | 1269 (9.7) | | | 232 (16.9) | <.001 | |
| Self-reported OH (fair/poor) | | 1615 (11.0) | | 1385 (10.6) | | | 230 (16.9) | <.001 | |
| Flossing <= 2 times per week | | 2671 (18.2) | | 2281 (17.4) | | | 390 (28.4) | <.001 | |
| Bone loss around teeth | | 2884 (19.7) | | 2627 (20.8) | | | 257 (19.7) | 0.35 | |
| Scaling and root planing | | 3426 (23.4) | | 3130 (24.1) | | | 296 (22.0) | 0.09 | |
| History of periodontitis | | 1072 (7.3) | | 963 (7.5) | | | 109 (8.2) | 0.39 | |

^a^ Data are presented as mean (standard deviation) for continuous variables; and n (%) for categorical variables.

^b^ P-values are from chi-square test for categorical variables or t-tests for continuous variables comparing groups.

**Abbreviations:** BMI, body-mass-index; OH, oral health.

Suppl. Table S5 Distribution of genetic information of the identified shared loci among the WGHS women based on the confirmed type 2 diabetes (T2D)

|  | Candidacy | Gene | Total | No T2D | Confirmed T2D | ***P*** ^b^ | |
| --- | --- | --- | --- | --- | --- | --- | --- |
| N |  |  | 14663 | 13271 (91) | 1392 (9) |  | |
| rs149290349_a_0 | perio11 | ZFP36L2 | 12539 (85.5) | 11320 (85.3) | 1219 (87.6) | 0.066 | |
| rs6711375_a_0 | perio11 | -- | 1556 (10.6) | 1427 (10.8) | 129 (9.3) | 0.19 | |
| rs4376068_a_0 | t2d_bmd | IGF2BP2 | 1536 (10.5) | 1371 (10.3) | 165 (11.9) | 0.014 | |
| rs4328980_a_0 |  | WFS1 | 6496 (44.3) | 5844 (44.0) | 652 (46.8) | 0.12 | |
| rs9348440_t_0 |  | CDKAL1 | 11219 (76.5) | 10164 (76.6) | 1055 (75.8) | 0.68 | |
| rs1265758_a* | perio11 | TSBP1,  TSBP1-AS1 | -- | -- | -- | -- | |
| rs2010390_a_0 | perio11 | LOC101929128 | 7206 (49.1) | 6536 (49.3) | 670 (48.1) | 0.72 | |
| rs13266634_t_0 |  | SLC30A8,  LOC105375716 | 7056 (48.1) | 6374 (48.0) | 682 (49.0) | 0.52 | |
| rs7020996_t_0 |  | -- | 10303 (70.3) | 9297 (70.1) | 1006 (72.3) | 0.21 | |
| rs11257655_t_0 |  | -- | 9327 (63.6) | 8457 (63.7) | 870 (62.5) | 0.11 | |
| rs61875362_t_0 |  | -- | 6145 (41.9) | 5565 (41.9) | 580 (41.7) | 0.71 | |
| rs12255678_t_0 | perio11 | TCF7L2 | 849 (5.8) | 755 (5.7) | 94 (6.8) | 0.001 | |
| rs75933965_a_0 | t2d_bmd | TCF7L2 | 12792 (87.2) | 11609 (87.5) | 1183 (85.0) | 0.028 | |
| rs10770140_t_0 | perio11 | TH, MIR4686 | 2109 (14.4) | 1873 (14.1) | 236 (17.0) | 0.013 | |
| rs3200401_t_0 | perio11 | MALAT1, MASCRNA,  TALAM1 | 9453 (64.5) | 8579 (64.6) | 874 (62.8) | 0.083 | |
| rs77464186_a_0 | t2d_bmd | ARAP1 | 347 (2.4) | 326 (2.5) | 21 (1.5) | 0.06 | |
| rs76895963_t_0 | 2d_bmd | CCND1,  CCND2-AS1 | 48 (0.3) | 1 (0) | 47 (3.4) | 0.52 | |
| rs17522122_t_0 | perio11, perio_t2d | AKAP6 | 4109 (28.0) | 3742 (28.2) | 367 (26.4) | 0.15 |  |
| rs8047395_a_0 | perio11 | FTO | 3503 (23.9) | 3203 (24.1) | 300 (21.6) | 0.071 |  |
| rs665268_a_0 | perio11 | MLX | 1087 (7.4) | 971 (7.3) | 116 (8.3) | 0.15 |  |
| rs2546494_a_0 | perio11 | LOC105371814 | 3447 (23.5) | 3127 (23.6) | 320 (23.0) | 0.86 |  |

^a^ Data are presented as n (%) for those with 0 allele as presented.

^b^ P-values are from chi-square test for categorical variables comparing groups.

Candidacy for common loci annotation: perio_t2d, SNP identified with H4 probability of T2D/PerioLT colocalization >0.5; perio11, SNP identified from the MTAG analysis and with the original GWAS p_perioLT_<0.1: t2d_bmd, additional SNP identified with H4 probability of T2D/BMD colocalization >0.7 or higher H3 probability of 2 different lead SNPs in T2D/BMD; *, rs1265758 was not available in the WGHS data.

All SNPs are followed by the effect allele with the 0 mldose information provided in the table.

Suppl. Table S6 Clinical dental characteristics related to responses of OHQs from a limited subset of WGHS women with retrieved dental records

|  | **N** | **Total NoT** |  | **Ant. NoT** |  | **Post. No.T** |  |
| --- | --- | --- | --- | --- | --- | --- | --- |
| ***Self_OH*** |  | mean (sd) | Pv ^a^ | mean (sd) | Pv ^a^ | mean (sd) | Pv ^a^ |
| Excellent-Good | 54 | 25.50 (3.9) | 0.005 | 11.52 (1.2) | 0.0004 | 13.98 (3.2) | 0.02 |
| Fair-Poor | 9 | 19.11 (6.4) |  | 9.44 (2.5) |  | 9.67 (4.9) |  |
| ***Dental visit*** |  |  |  |  |  |  |  |
| <= 1 year | 62 | 24.61 (4.8) | 0.48 | 11.21 (1.6) | 0.52 | 13.40 (3.8) | 0.33 |
| > 1 year | 1 | 23 |  | 12 |  | 11 |  |
| ***Prophylaxis*** |  |  |  |  |  |  |  |
| >= once per year | 56 | 25.21 (4.3) | 0.005 | 11.41 (1.3) | 0.05 | 13.80 (3.6) | 0.02 |
| < once per year | 7 | 19.57 (6.0) |  | 9.71 (2.8) |  | 9.86 (3.6) |  |
| ***BL around teeth*** |  |  |  |  |  |  |  |
| No | 30 | 26.03 (3.7) | 0.01 | 11.87 (0.3) | 0.05 | 14.17 (3.6) | 0.006 |
| Yes | 24 | 23.00 (5.7) |  | 10.67 (2.1) |  | 12.33 (3.8) |  |
| ***Self_PD*** |  |  |  |  |  |  |  |
| No | 47 | 24.89 (4.6) | 0.22 | 11.34 (1.4) | 0.10 | 13.55 (3.8) | 0.21 |
| Yes | 14 | 23.36 (5.5) |  | 10.71 (2.1) |  | 12.64 (3.9) |  |
| ***Flossing*** |  |  |  |  |  |  |  |
| > 2 per week | 54 | 24.28 (5.1) | 0.44 | 11.13 (1.7) | 0.44 | 13.15 (4.0) | 0.45 |
| < 2 per week | 9 | 26.44 (1.8) |  | 11.78 (0.4) |  | 14.67 (1.6) |  |
| ***SCRP*** |  |  |  |  |  |  |  |
| No | 25 | 24.32 (4.8) | 0.71 | 11.40 (1.2) | 0.77 | 12.92 (4.2) | 0.70 |
| Yes | 37 | 24.81 (4.9) |  | 11.11 (1.8) |  | 13.70 (3.6) |  |

^a^ P-values are from t-tests for continuous variables comparing groups.

**Abbreviations:** NoT, number of teeth; Ant, anterior; Post, posterior; BL, bone loss.

Supplementary Table S7 Significant genetic associations with responses to oral health questions (OHQs) adjusting for more traditional risk factors

|  | Dental Visit > 1 year ago | | Prophylaxis < 1 per year | | Floss <= 2 per week | | Bone loss around teeth | |
| --- | --- | --- | --- | --- | --- | --- | --- | --- |
|  | **OR (95%CI)** | **p** | **OR (95%CI)** | **p** | **OR (95%CI)** | **p** | \| **OR (95%CI)** \| **Pv** \| \| --- \| --- \| | **p** |
| ***Colocalized*** |  |  |  |  |  |  |  |  |
| rs17522122_t | 0.94 (0.86-1.03) | 0.20 | 0.98 (0.89-1.08) | 0.75 | 0.92 (0.86-0.98) | 0.01 | 1.03 (0.97-1.1) | 0.34 |
| ***Suggestive*** |  |  |  |  |  |  |  |  |
| rs3200401_t | 1.04 (0.93-1.16) | 0.52 | 1.05 (0.93-1.18) | 0.47 | 1.08 (0.99-1.17) | 0.08 | 0.98 (0.9-1.07) | 0.67 |
| rs149290349_a | 0.84 (0.7-1.01) | 0.07 | 0.93 (0.77-1.11) | 0.43 | 1.08 (0.95-1.22) | 0.23 | 1 (0.89-1.13) | 0.94 |
| rs6711375_a | 0.94 (0.86-1.04) | 0.24 | 0.9 (0.81-0.99) | 0.03 | 1 (0.93-1.08) | 0.96 | 0.98 (0.92-1.05) | 0.64 |
| rs2010390_a | 0.96 (0.86-1.07) | 0.44 | 1.05 (0.94-1.18) | 0.40 | 0.96 (0.89-1.04) | 0.35 | 1.07 (0.99-1.15) | 0.11 |
| rs12255678_t | 1.03 (0.92-1.14) | 0.63 | 1.08 (0.97-1.21) | 0.17 | 1.04 (0.96-1.13) | 0.31 | 1.01 (0.94-1.09) | 0.74 |
| rs10770140_t | 1.03 (0.94-1.14) | 0.49 | 1.12 (1.02-1.24) | 0.02 | 1.02 (0.95-1.09) | 0.67 | 0.95 (0.89-1.01) | 0.1 |
| rs8047395_a | 0.97 (0.89-1.06) | 0.53 | 0.98 (0.89-1.07) | 0.61 | 0.95 (0.89-1.02) | 0.13 | 1.09 (1.02-1.16) | 0.01 |
| rs665268_a | 0.94 (0.85-1.04) | 0.25 | 0.92 (0.83-1.03) | 0.13 | 0.96 (0.89-1.03) | 0.24 | 1.02 (0.95-1.1) | 0.61 |
| rs2546494_a | 0.94 (0.86-1.03) | 0.17 | 0.93 (0.84-1.02) | 0.12 | 0.98 (0.92-1.05) | 0.56 | 1.01 (0.94-1.07) | 0.87 |
| ***Additional*** |  |  |  |  |  |  |  |  |
| rs4376068_a | 0.98 (0.89-1.08) | 0.63 | 1.03 (0.93-1.15) | 0.52 | 0.94 (0.87-1.01) | 0.08 | 1.02 (0.96-1.1) | 0.50 |
| rs75933965_a | 1.05 (0.87-1.25) | 0.60 | 1.02 (0.83-1.23) | 0.88 | 1.2 (1.06-1.37) | 0.005 | 0.95 (0.83-1.08) | 0.46 |
| rs77464186_a | 0.86 (0.76-0.97) | 0.01 | 0.82 (0.72-0.93) | 0.002 | 0.97 (0.88-1.06) | 0.49 | 0.99 (0.91-1.08) | 0.84 |
| rs76895963_t | 0.79 (0.56-1.13) | 0.18 | 1.02 (0.69-1.58) | 0.93 | 0.75 (0.58-0.98) | 0.03 | 1.21 (0.91-1.62) | 0.20 |

**Models:** The associations to different oral health question responses were assessed with multivariate logistic regression using the genetic variables as the independent variables, adjusting for age at the time of OHQ responses, baseline information: smoking (3 groups), BMI, hypercholesterolemia, hypertension, and updated osteoporosis, confirmed diabetes status, and 10 genomic eigenvectors. **Abbreviations:** OR, odds ratio; CI, confidence interval. **Allele:** effect allele shown after each variant.

Supplementary Table S8 Non-significant genetic associations with responses to oral health questions (OHQs)

|  | Self-reported fair/poor OH | | Self-reported PD | | SCRP | |
| --- | --- | --- | --- | --- | --- | --- |
|  | **OR (95%CI)** | **Pv** | **OR (95%CI)** | **Pv** | **OR (95%CI)** | **Pv** |
| ***Colocalized*** |  |  |  |  |  |  |
| rs17522122 | 1.04 (0.97-1.13) | 0.249 | 1.09 (1-1.19) | 0.062 | 0.99 (0.94-1.05) | 0.837 |
| ***Suggestive*** |  |  |  |  |  |  |
| rs3200401 | 0.93 (0.85-1.02) | 0.148 | 1.05 (0.94-1.17) | 0.41 | 1.03 (0.96-1.1) | 0.442 |
| rs149290349 | 0.93 (0.8-1.07) | 0.322 | 0.95 (0.8-1.13) | 0.577 | 1 (0.9-1.11) | 0.931 |
| rs6711375 | 0.96 (0.89-1.04) | 0.286 | 1.04 (0.95-1.15) | 0.37 | 1.02 (0.96-1.08) | 0.47 |
| rs2010390 | 1.02 (0.94-1.12) | 0.593 | 0.98 (0.88-1.09) | 0.689 | 1.02 (0.96-1.09) | 0.514 |
| rs12255678 | 1.09 (1-1.19) | 0.05 | 1.09 (0.98-1.21) | 0.104 | 1.01 (0.95-1.08) | 0.662 |
| rs10770140 | 0.99 (0.92-1.07) | 0.805 | 0.96 (0.88-1.05) | 0.37 | 0.97 (0.91-1.02) | 0.229 |
| rs8047395 | 1.01 (0.94-1.09) | 0.752 | 1.01 (0.92-1.11) | 0.819 | 1.04 (0.98-1.1) | 0.171 |
| rs665268 | 1 (0.92-1.09) | 0.998 | 0.96 (0.87-1.06) | 0.446 | 1.04 (0.98-1.11) | 0.232 |
| rs2546494 | 0.99 (0.92-1.07) | 0.788 | 1.04 (0.95-1.14) | 0.393 | 1.02 (0.97-1.08) | 0.401 |
| ***Additional*** |  |  |  |  |  |  |
| rs4376068 | 1.04 (0.96-1.13) | 0.336 | 0.98 (0.9-1.08) | 0.75 | 1.02 (0.96-1.08) | 0.53 |
| rs75933965 | 0.96 (0.82-1.11) | 0.566 | 1.05 (0.88-1.25) | 0.579 | 1 (0.89-1.11) | 0.957 |
| rs77464186 | 0.97 (0.88-1.08) | 0.593 | 1.06 (0.94-1.21) | 0.331 | 1 (0.93-1.08) | 0.96 |
| rs76895963 | 0.79 (0.61-1.06) | 0.108 | 0.83 (0.6-1.17) | 0.262 | 0.92 (0.74-1.15) | 0.464 |

**Models:** The associations to different oral health question responses were assessed with multivariate logistic regression using the genetic variables as the independent variables, adjusting for age at the time of OHQ responses, baseline information smoking (3 groups), and 10 genomic eigenvectors. **Abbreviations:** OH, oral health; PD, periodontal disease; SCRP, scaling and root planing; OR, odds ratio; CI, confidence interval.

Suppl. Table S9 Summary of information from PheWAS, GWAS catalog and eQTLs for selected candidate loci (accessed on 3/16/2022)

| **SNP** | **Chr:Pos** | **Name** | **# of**  **PheWAS (p<5e^-08^)** | **# of PubMed**  **# of GWAS catalog** | **# eQTL** |
| --- | --- | --- | --- | --- | --- |
| **Colocalized** |  |  |  |  |  |
| rs17522122  [floss] | 14:33302882 | AKAP6  (3’ UTR) | **66 PheWAS**  13 body measures  28 body fat related  6 cognitive intelligences  6 body impedance  4 body mass related  2 spherical power  2 walking pace  1 eyesight  1 basal metabolic rate  1 dexterity  1 household income | **4 PubMed citation**  1 Glioma  3 Cognitive performance  **19 GWAS catalog**  1 SBP  9 BMI  1 Prostate cancer  3 Type 2 diabetes  3 Physical activities  1 Cognitive function | **0 eQTL** |
| **Suggestive** |  |  |  |  |  |
| rs6711375  [prophy] | 2:161090873 | -- | **44 PheWAS**  5 body measures  23 body fat related  1 secretory phospholipase A2 receptor  2 membrane neuropathy  **Blood cells related:**  1 leukocyte  3 lymphocyte  2 reticulocyte | **0 PubMed citation**  **0 GWAS catalog** | **4 eQTL**  ENSG00000054219 (LY75)  ENSG00000123636 (BAZ2B)  ENSG00000241399 (CD302)  ENSG00000224152 (BAZ2B, antisense) |
| rs8047395  [bone loss] | 16:53798523 | FTO | **229 PheWAS**  17 body mass index  39 body measures  38 body fat related  6 bone mineral density  10 body impedance  23 body mass related  11 obesity  16 type 2 diabetes  2 basal metabolic rate  2 menarche  7 sleep disorder  5 glucose homeostasis  6 breast cancer  7 cholesterol related  4 chronotype  6 high blood pressure  13 dietary and nutritional related  1 alanine aminotransferase  1 c-reactive protein  1 SHBG  1 sodium in urine  2 cystatin c  2 urate  1 total testosterone  1 waking pace  **Blood cells related:**  1 leukocyte  1 red blood cells  1 neutrophil  4 reticulocyte | **12 PubMed citation**  BMI, FTO and thyroid cancer  CNS Tumor  Glioma  BMI and diabetes  Metabolic syndrome  Glucose homeostasis  Obesity  Cancer risk  **0 GWAS catalog** | **0 eQTL** |
| **Additional** |  |  |  |  |  |
| rs75933965  [floss] | 10:114749421 | TCF7L2 | **29 PheWAS**  2 body fat related  21 diabetes  2 metformin  2 HbA1c  2 glucose  1 gliclazide | **0 PubMed citation**  **0 GWAS catalog** | **0 eQTL** |
| rs77464186  [dental visit, prophy] | 11:72460398 | ARAP1 | **34 PheWAS**  2 body fat related  2 body mass related  18 diabetes  2 HbA1c  2 glucose  2 metformin  1 education  **Blood cells related:**  1 platelet | **0 PubMed citation**  **5 GWAS catalog**  2 Medication for diabetes  1 fasting glucose  2 type 2 diabetes | **3 eQTL**  ENSG00000214530 (STARD10)  ENSG00000245148 (ARAP-AS2)  ENSG00000186635 (ARAP1) |
